# The Relationship Between Physical Activity and Interpersonal Distress Among High School Students: A Moderated Mediation Model

**DOI:** 10.3390/bs15091155

**Published:** 2025-08-25

**Authors:** Hanwen Chen, Tianci Lu, Baole Tao, Jun Yan

**Affiliations:** College of Physical Education, Yangzhou University, Yangzhou 225127, China; dx120230091@stu.yzu.edu.cn (H.C.); dx120240094@stu.yzu.edu.cn (T.L.); dx120190064@yzu.edu.cn (B.T.)

**Keywords:** physical activity, interpersonal distress, regulatory emotional self-efficacy, psychological resilience, high school students

## Abstract

This study aims to explore the relationship between physical activity and interpersonal distress among high school students and its mechanism of action. Based on triadic reciprocal determinism, social cognitive theory, and the protective factor–protective factor Model, a cross-sectional study was conducted on 2072 high school students using the International Physical Activity Questionnaire Short Version (IPAQ-S), the Regulatory Emotional Self-Efficacy Scale, the Adolescent Psychological Resilience Scale, and the Interpersonal Relationship Comprehensive Diagnosis Scale. The results showed that (1) physical activity has a significant adverse predictive effect on interpersonal distress among high school students. (2) Physical activity can not only directly predict interpersonal distress among high school students, but also indirectly predict interpersonal distress through the mediating effect of regulatory emotional self-efficacy. (3) Psychological resilience plays a moderating role in the relationship between emotional regulation self-efficacy, and interpersonal distress. For high school students with low psychological resilience, emotional regulation self-efficacy has a more significant predictive effect on interpersonal distress; however, for high school students with high psychological resilience, the moderating effect is not significant.

## 1. Introduction

Adolescence represents a pivotal period of transformation in social relationships and a critical stage in the development of self-identity. The construction of self-identity occurs within specific interpersonal contexts, where the need for self-integration is reflected in adolescents’ desire to establish and sustain stable relationships with others (Xue et al., 2021). Positive interpersonal relationships not only facilitate academic achievement but also enhance social competence, self-awareness, and mental health. Nevertheless, interpersonal relationships often pose challenges for adolescents due to various factors. Many struggle to form satisfying relationships and consequently experience interpersonal distress (Kiuru et al., 2020). Interpersonal distress refers to negative emotions—such as anxiety, loneliness, and depression—arising from conflicts in values, inadequate communication skills, or strained relationships (Kehl et al., 2021; X. Li, 2024). Surveys indicate that the prevalence of interpersonal distress among Chinese adolescents ranges from 35.0% to 46.5% (Y. Zhang et al., 2023; M. Li et al., 2023). Such distress not only undermines academic performance and school adjustment but is also closely associated with difficulties in emotion regulation, depression, and problematic behaviors (J. Li et al., 2020; Rose-Clarke et al., 2021). Given that middle school is a critical stage for social development, examining the factors influencing students’ interpersonal relationships is essential for promoting academic engagement, social adjustment, and psychological well-being.

### 1.1. The Relationship Between Physical Activity and Interpersonal Distress

Given the strong association between interpersonal distress, mental health, and subjective well-being, researchers have increasingly examined its potential predictors. Lack of physical activity may be considered one of the predictors of interpersonal distress. Evidence suggests that when individuals struggle to establish or maintain satisfying interpersonal relationships in academic contexts, engaging in regular physical activity can foster conditions conducive to improving these relationships (Kim et al., 2021). According to social cognitive theory, and particularly its central principle of triadic reciprocal determinism, behavior, personal factors, and environmental influences are interdependent and mutually reinforcing (Bandura, 1978). Behavior provides a pathway through which individuals interact with their environment, while environmental influences exert reciprocal effects through behavioral engagement (Bandura, 1986). More specifically, environmental affordances are realized when activated by corresponding behavioral patterns.

Regular physical activity significantly enhances an individual’s physical and mental health, as well as subjective well-being. It helps high school students alleviate stress, improve their emotional state (e.g., reduce anxiety and depression), and boost self-efficacy (J. Li et al., 2022; G. Zhang et al., 2024). These positive internal changes form a crucial foundation for improving interpersonal interactions and fostering more positive relationships. Notably, participation in group physical activities offers a unique opportunity for the “externalization” of these internal changes and the development of interpersonal relationships. Such activities create a natural setting for high-frequency interactions, shared goals (e.g., completing exercise tasks together), and social communication (M. Li et al., 2025). In this environment, individuals can practice social skills, express emotions, and share positive experiences with peers (e.g., friends, teammates) during physical activities (Zhu & Shu, 2022). By achieving exercise goals cooperatively, individuals not only reinforce the positive emotions and sense of efficacy derived from physical activity but also engage in constructive social behaviors (such as cooperation, support, and encouragement). Thus, regular physical activity not only provides an internal foundation for positive social interactions by improving psychological well-being but also translates this foundation into actual interpersonal connections and positive social behaviors through structured social interaction. This synergy between internal improvement and environmental interaction fosters the development of a broader and more supportive interpersonal network, thereby effectively reducing interpersonal distress.

### 1.2. The Mediating Role of Regulatory Emotional Self-Efficacy

Social cognitive theory emphasizes that self-efficacy—an individual’s belief in their ability to perform behaviors to achieve desired outcomes—is a core determinant of sustained behavioral change and adaptation (Bandura, 1998). In complex interpersonal contexts, regulatory emotional self-efficacy, a domain-specific aspect of self-efficacy, highlights an individual’s perceived ability to manage emotional states (G. V. Caprara et al., 2008). Bandura et al. (2003) further suggest that regulatory emotional self-efficacy involves confidence in recognizing one’s own emotions, understanding others’ feelings, and managing both positive and negative emotional expressions. Thus, regulatory emotional self-efficacy reflects confidence in recognizing, understanding, and regulating emotions, and may mediate the relationship between physical activity and alleviating adolescents’ interpersonal distress. Empirical evidence shows that individuals engaged in regular physical activity tend to exhibit higher levels of regulatory emotional self-efficacy (Xu & Du, 2021). Through these activities, individuals gain emotional regulation experience—both directly and by observing others—which enhances their emotional self-efficacy (Jiang et al., 2018). Consistent with triadic reciprocal determinism, behavior can shape internal factors such as self-perception, emotions, and attitudes (Bandura, 1986). Thus, physical activity not only strengthens self-efficacy but also reinforces emotional regulation confidence, allowing adolescents to manage emotional events more proactively and effectively (G. Zhang et al., 2024).

In addition, social cognitive theory highlights the dynamic interplay between self-efficacy and peer interaction. The theory posits that individuals’ behaviors are shaped by their cognitive appraisals of personal abilities, with higher levels of self-efficacy linked to more adaptive interpersonal interactions (Bandura, 1998). Bandura further emphasizes that self-efficacy directly influences effort, persistence in the face of setbacks, and coping strategies in social contexts (Bandura, 1999). Empirical studies corroborate these claims, showing that individuals with greater emotional self-efficacy tend to demonstrate more constructive patterns of interpersonal interaction, as they display higher confidence and adopt a proactive stance in social situations (Rashid et al., 2024). Taken together, these findings suggest that regulatory emotional self-efficacy may function as a protective factor against interpersonal distress. Accordingly, it may mediate the relationship between physical activity and interpersonal distress among adolescents.

### 1.3. The Moderating Role of Psychological Resilience

Other factors may influence the mediating role of regulatory emotional self-efficacy. Psychological resilience refers to the intrinsic strengths individuals display in adversity and is a basic quality for coping with stress and a key indicator of mental health (Masten, 2019). As an intrinsic protective factor, it helps individuals overcome the adverse effects of multiple risks (Sisto et al., 2019). From the perspective of positive psychology, psychological resilience is considered a crucial trait of adolescent socialization, predicting positive life outcomes, supporting daily adaptation, and alleviating interpersonal distress (Lin et al., 2024). Specifically, adolescents with high levels of psychological resilience are more likely to use adaptive cognitive strategies such as positive reappraisal and proactive problem-solving behaviors to cope with stress and frustration in interpersonal interactions, making them more flexible in interpersonal relationships, able to view various interpersonal events more positively, and effectively deal with interpersonal conflicts, thereby significantly reducing the occurrence of interpersonal distress (Zhao et al., 2020).

The protective–protective model’s promoting hypothesis suggests that different protective factors may have interactive effects in predicting outcome variables (Fergus & Zimmerman, 2005). This model suggests that the strength or direction of the influence of one protective factor (regulatory emotional self-efficacy) on its outcome variable (interpersonal distress) may be enhanced by the level of another protective factor (psychological resilience). Empirical evidence shows that psychological resilience directly counteracts or compensates for adverse effects of risks (Sun et al., 2025). This study examines the interaction between psychological resilience and regulatory emotional self-efficacy in buffering interpersonal distress among high school students. Specifically, psychological resilience, as a general coping resource, may enhance the protective effect of regulatory emotional self-efficacy, a more specific emotional management resource, on interpersonal distress. This not only enriches the protective–protective model but also provides new insight into the synergy of psychological resources in addressing interpersonal distress among high school students. We argue that psychological resilience may moderate the relationship between regulatory emotional self-efficacy and interpersonal distress, with higher resilience amplifying the protective effect.

### 1.4. Current Study Aims and Hypotheses

**Figure 1:**
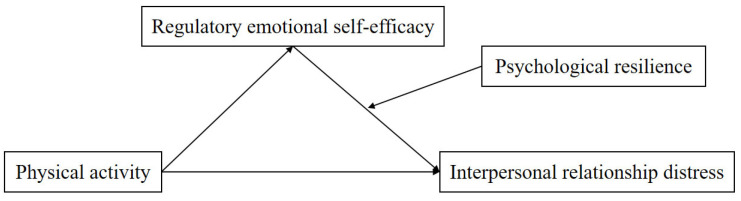
Assumed moderated mediation model.

Although studies have explored the benefits of physical activity on adolescent mental health and the roles of regulatory emotional self-efficacy or psychological resilience, there is limited research on how physical activity predicts interpersonal distress in high school students through regulatory emotional self-efficacy and how psychological resilience modulates this pathway. Specifically, the interaction between regulatory emotional self-efficacy and psychological resilience in the mechanism by which physical activity alleviates interpersonal distress has not been fully explored. Based on triadic reciprocal determinism and social cognitive theory, this study proposes a moderated mediation model with high school students to examine the mediating role of regulatory emotional self-efficacy between physical activity and interpersonal distress, and the moderating role of psychological resilience. This study aims to provide insights into the psychological mechanisms underlying the relationship between physical activity and interpersonal distress and offer practical recommendations for alleviating interpersonal distress among high school students. This study proposes the following hypotheses (Figure 1): H1. Physical activity can negatively predict interpersonal distress among high school students; H2. Emotional regulation and self-efficacy mediate the relationship between physical activity and interpersonal distress among high school students; H3. Psychological resilience moderates the relationship between emotional regulation self-efficacy and interpersonal distress among high school students.

## 2. Methods

### 2.1. Participants

We selected four regions of Jiangsu Province: northern, central, and southern Jiangsu, and Nanjing City. From each region, one urban public high school and one rural public high school were chosen, yielding a total of eight schools. High school students from these schools served as participants. A total of 2354 questionnaires were distributed, of which 2075 valid responses were retained after excluding incomplete or randomly answered questionnaires, resulting in an effective response rate of 88.15%. The sample consisted of first- and second-year students (M = 16.45, SD = 0.71). Third-year students were not included because they were preparing for the college entrance examination, and to avoid interference, no questionnaires were administered to them. Table 1 presents the descriptive statistics of the participants’ socio-demographic characteristics. The sample included 1071 males (51.6%) and 1004 females (48.4%), with an average age of 16.45 years. Of these, 1011 students (48.7%) were in the first year of high school and 1064 (51.3%) in the second year. By region, 537 students (25.9%) were from northern Jiangsu, 567 (27.3%) from central Jiangsu, 486 (23.4%) from southern Jiangsu, and 485 (23.4%) from Nanjing City.

### 2.2. Procedure

This cross-sectional study was conducted in Jiangsu Province, China, with approval from the Ethics Committee of Yangzhou University Medical College (YXYLL-2022-121). Questionnaires were administered offline with the support of school administrators. Participants completed the survey on site under standardized instructions and returned the forms immediately. Informed consent was obtained from all students prior to participation. Inclusion criteria were (1) no serious physical illness in the recent past and (2) no diagnosed mental disorders or cognitive impairments. Returned questionnaires were screened, and those with uniform responses throughout or incomplete answers were excluded.

### 2.3. Research Tools

#### 2.3.1. International Physical Activity Questionnaire Short Version (IPAQ-S)

Physical activity refers to any bodily movement produced by skeletal muscles that results in energy expenditure. In this study, physical activity was operationally defined as the total volume of habitual physical activity, measured using the Chinese version of the International Physical Activity Questionnaire Short Version (IPAQ-S; Qu & Li, 2004). The IPAQ-S assesses self-reported physical activity levels through seven items covering three domains: walking, moderate-intensity activities, and vigorous-intensity activities. It quantifies physical activity by calculating the total metabolic equivalent (MET-min/week), where energy expenditure values are assigned to each activity type based on established MET coefficients. This standardized metric serves as our empirical proxy for participants’ overall physical activity levels. As a widely validated instrument for population studies, the IPAQ-S has demonstrated good reliability and validity in Chinese adolescents (B. Hu et al., 2015). For analysis, we summed the MET-min/week values across all activity domains to derive a total physical activity score for each participant.

#### 2.3.2. Adolescent Psychological Resilience Scale

The adolescent Psychological Resilience Scale (PR) developed by Chinese scholars Hu Yueqin and Gan Yiqun was used (Y. Hu & Gan, 2008). The scale consists of 27 items divided into five dimensions: goal focus, emotional control, positive cognition, family support, and interpersonal assistance. The scale uses a 5-point Likert scale, with higher scores indicating better psychological resilience.

#### 2.3.3. Regulatory Emotional Self-Efficacy Scale

The Chinese version of the Caprara et al. revised Regulatory Emotional Self-Efficacy Scale (RESE) by Chinese scholars Wen Shufeng and Tang Dongling was used (Wen et al., 2009). It consists of three dimensions: positive emotional self-efficacy (POS), depressive emotional self-efficacy (DES), and anger emotional self-efficacy (ANG), with a total of 12 items. A 5-point scoring method is used, with higher scores representing higher emotional regulation ability.

#### 2.3.4. Interpersonal Relationship Comprehensive Diagnostic Scale

The Interpersonal Relationship Comprehensive Diagnostic Scale (IRCD) developed by Zheng Richang et al. was used (Zheng, 1999). This scale primarily diagnoses and measures interpersonal relationships from four aspects: interpersonal friendship, interpersonal communication, interpersonal interaction, and interaction with the opposite sex. The scale consists of 28 items, each answered with either “yes” or “no.” A total score of 0–8 indicates no interpersonal distress, a score of 9–14 reflects mild interpersonal distress, and a score of 15–28 suggests more severe interpersonal distress. Therefore, a higher total score indicates a higher level of interpersonal distress experience.

### 2.4. Data Processing

This study employed SPSS 26.0 (IBM Corp., Armonk, NY, USA) and Mplus 8.2 (Muthén & Muthén, Los Angeles, CA, USA) for statistical analysis. First, descriptive statistics were conducted on the participants, including means, standard deviations, and percentages to describe the socio-demographic characteristics of the participants and the characteristics of the study variables. Second, Cronbach’s alpha coefficient and confirmatory factor analysis (CFA) were used to ensure the reliability and validity of the scales. Subsequently, Harman’s one-factor test and variance inflation factor (VIF) were employed to assess common method bias. Fourth, correlation analysis was conducted to establish preliminary relationships among the variables. Finally, to test the moderation and mediation model (Figure 1), we used Hayes’ PROCESS macro (version 4.1) for SPSS analysis (Hayes, 2022). The computational process employed path analysis based on ordinary least squares regression, combined with bootstrap resampling. This method is robust for non-normal distributions and provides higher statistical power in complex models compared to traditional methods. Research indicates that Hayes’ PROCESS macro model effectively assesses mediating and moderating effects and enhances the robustness of results through bootstrap sampling (Lu et al., 2025). In this study, bootstrap sampling was applied to 10,000 resampled data points, and the significance of path coefficients was tested using 95% confidence intervals with bias correction.

## 3. Results

### 3.1. Measurement Reliability and Validity

All instruments demonstrated adequate psychometric properties in the current sample. As shown in Table 2, the Cronbach’s alpha coefficients for each scale ranged from 0.852 to 0.907, all exceeding the acceptable standard of 0.70; the fit indices from confirmatory factor analysis also met the requirements (*χ*^2^/df < 6, CFI > 0.85, TLI > 0.85, RMSEA < 0.08), indicating that the measurement model fits the data well. Although some fit indices (such as CFI and TLI) are slightly below the ideal standard of 0.90, considering the large number of scale items and the large sample size, these indices are still within an acceptable range (L. Hu & Bentler, 1999).

### 3.2. Common Method Bias Test and Collinearity Test

Since this study only used self-reporting methods to collect data, the results may be affected by common method bias. The Harman single-factor test was used to examine common method bias (Zhou & Long, 2004). Exploratory factor analysis was performed on all items in the Adolescent Psychological Resilience Scale, Regulatory Emotional Self-Efficacy Scale, and Interpersonal Relationship Comprehensive Diagnosis Scale using SPSS 26.0 (IBM Corp., Armonk, NY, USA). The results showed that, according to the unrotated factor analysis, a total of 13 factors with characteristic roots greater than 1 were extracted, and the maximum factor explained variance was 18.738%, which was below the critical standard of 40%. Therefore, there was no obvious common method bias in this study. Collinearity tests were conducted on the independent variables, mediating variables, and moderating variables. The results showed that the tolerance intervals for all variables ranged from 0.669 to 0.997, exceeding 0.1, and the VIF values ranged from 1.024 to 1.494, all below 10, indicating no severe collinearity among the variables.

### 3.3. Descriptive Statistics and Correlation Analysis

The results showed that the rate of interpersonal distress among high school students was 48%, of which 33.5% were mildly distressed and 14.5% were severely distressed. The results of the independent sample t-test showed (Table 3) that there were significant differences between genders in terms of physical activity and regulatory emotional self-efficacy. Analysis of variance showed that there were significant regional differences in physical activity, regulatory emotional self-efficacy, psychological resilience, and interpersonal distress. There were significant differences in family type in terms of emotional regulation self-efficacy, psychological resilience, and interpersonal distress. Therefore, subsequent studies included gender, region, and family type as control variables in the analysis. Table 4 shows the correlation between each variable. There was a significant negative correlation between interpersonal distress and high school students’ physical activity, emotional regulation self-efficacy, and psychological resilience; there was a significant positive correlation between physical activity, emotional regulation self-efficacy, and psychological resilience.

### 3.4. The Relationship Between Physical Activity and Interpersonal Distress: Testing a Moderated Mediation Model

The results of the correlation analysis met the statistical requirements for further testing of the mediating effect. All research variables in this study were standardized, and gender, region, and family type were used as control variables (region and family type are treated as dummy variables). Model 4 in the PROCESS macro (version 4.1) developed by Hayes was used to test the mediating effect of regulatory emotional self-efficacy (Hayes, 2022). The results (Table 5 and Table 6) show that after controlling for the gender, region, and family type of high school students, the direct path coefficient between physical activity and interpersonal distress among high school students (β = −0.075, t = −3.535, *p* < 0.01), with a direct effect value of −0.075 and a bootstrap 95% confidence interval of [−0.116, −0.033]. The path coefficients of physical activity and regulatory emotional self-efficacy (β = 0.122, t = 5.534, *p* < 0.001), and regulatory emotional self-efficacy and interpersonal distress (β = −0.355, t = −16.986, *p* < 0.001) were significant, with a mediation effect value of −0.043 and a bootstrap 95% confidence interval of [−0.061, −0.027]. Therefore, regulatory emotional self-efficacy plays a partial mediating role in the relationship between physical activity and interpersonal distress among high school students.

Second, Model 14 in the PROCESS macro (version 4.1) was used to test the moderated mediation model (Hayes, 2022). After controlling for gender, region, and family type, we used emotional regulation self-efficacy as the mediating model. The test results are shown in Table 7. After adding psychological resilience to the model, the interaction between emotional regulation self-efficacy and psychological resilience on interpersonal distress was significant (β = 0.042, t = 2.649, *p* < 0.01), indicating that psychological resilience moderated the relationship between emotional regulation self-efficacy and interpersonal distress.

**Figure 2:**
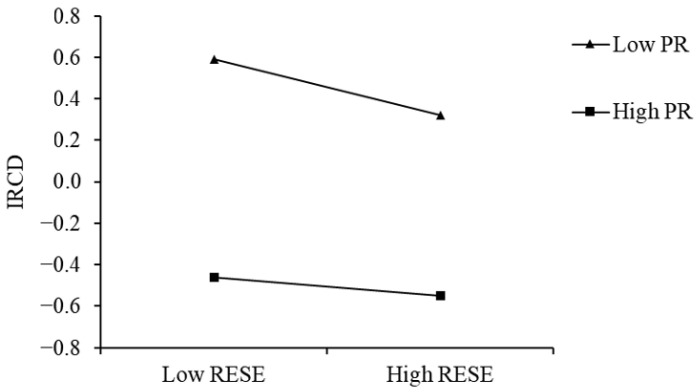
Interaction between regulatory emotional self-efficacy and psychological resilience on interpersonal distress.

In order to better explain the moderated mediation model, psychological resilience was divided into two groups, high psychological resilience and low psychological resilience, based on the addition or subtraction of one standard deviation. A simple slope test was used to examine the relationship between psychological resilience and regulatory emotional self-efficacy and interpersonal distress. The specific regulatory effects are shown in Figure 2 and Table 8. The results show that for high school students with low psychological resilience, emotional regulation self-efficacy can significantly predict interpersonal distress (β = −0.127, t = −4.756, *p* < 0.001), but for high school students with high psychological resilience, this prediction is not significant (β = −0.043, t = −1.488, *p* = 0.140).

## 4. Discussion

Based on triadic reciprocal determinism, social cognitive theory, and the protective–protective model, this study examined the path through which physical activity affects interpersonal distress by regulating regulatory emotional self-efficacy and explored the moderating role of psychological resilience in this process. The findings are as follows:

### 4.1. The Relationship Between Physical Activity and Interpersonal Distress Among High School Students

This study found that physical activity negatively predicts interpersonal distress among high school students. Higher levels of physical activity are associated with a lower likelihood of interpersonal distress. For high school students experiencing interpersonal distress, persistent negative interpersonal behavior can trigger maladaptive cognition, leading to depression and anxiety, which creates a vicious cycle (Mason et al., 2022). Based on triadic reciprocal determinism, physical activity, as an enjoyable and beneficial activity, plays a key role in intervening in this cycle (Bandura, 1986). Its mechanism lies in the interaction between behavior, internal factors, and the environment. First, physical activity improves emotional states and subjective pleasure by releasing neurotransmitters such as endorphins and dopamine (Mahindru et al., 2023), reversing negative perceptions stemming from interpersonal distress. Second, participating in structured or social physical activity can “activate” specific social environments. However, it is important to emphasize that the nature of this activated environment is critical to the developmental outcomes of high school students. Ideally, a well-designed, supportive, and inclusive group activity environment can be shaped into a relatively controlled, low-threat social setting. In such a positive environment, students can safely practice social skills, receive peer support, and obtain constructive feedback (Wu et al., 2024). Subsequently, they will gradually break their original pattern of social avoidance behavior and, in the process, enhance their sense of control and self-efficacy in social situations (N. Li et al., 2024). The positive internal state (improved emotion, increased motivation, and enhanced self-confidence) triggered by physical activity and the positive experience of a supportive environment, which in turn encourages individuals to engage in more proactive and positive interpersonal interactions in a wider range of social situations.

Therefore, under supportive social conditions, physical activity can initiate and maintain a virtuous triadic cycle by improving internal psychological states and shaping positive social environments. Within this dynamic cycle, the reciprocal reinforcement of behavior, individual factors, and environmental conditions fosters healthier interpersonal patterns, thereby mitigating loneliness, reducing social anxiety, and alleviating interpersonal distress (Nie et al., 2025b). This is consistent with the core principles of developmental psychology and behavioral intervention, which emphasize that early positive behavioral choices can generate a “snowball effect” with enduring positive impacts on the development of psychological variables (Bai & Yang, 2025). However, the initiation of this cycle is not absolute. Negative social factors (e.g., peer rejection, excessive pressure, bullying) in the group environment or bullying due to insufficient motor skills or poor fitness can undermine its effectiveness (García-Hermoso et al., 2020). Therefore, the success of physical activity, particularly group activities, in reducing interpersonal distress depends on the quality of the social environment and the inclusiveness of the activity design. Future research and practice should focus on optimizing the psychosocial environment in adolescent sports to maximize their benefits.

### 4.2. The Mediating Effect of Regulatory Emotional Self-Efficacy

This study found that regulatory emotional self-efficacy partially mediates the relationship between physical activity and interpersonal distress. First, physical activity significantly predicts regulatory emotional self-efficacy. Participation not only improves mood and relieves stress in the short term but also enhances the ability to cope with future emotional challenges (Martín-Rodríguez et al., 2024). From the perspective of triadic reciprocal determinism, physical activity is a behavior that provides individuals with direct mastery experiences. Actively engaging in and successfully managing the physical and emotional changes it induces represents the most powerful source of self-efficacy (Peng et al., 2025). Repeated successful experiences strengthen individuals’ belief that they can regulate physiological arousal and emotional states triggered by activities or external stressors, thereby enhancing their regulatory emotional self-efficacy. In addition, physical activity offers opportunities to observe peers’ coping strategies and emotional regulation in sports, while also providing social support (Nie et al., 2025a), further reinforcing confidence in one’s own emotional regulation abilities. According to social cognitive theory, self-efficacy beliefs profoundly shape cognition, motivation, emotions, and behavior (Bandura, 1998). High regulatory emotional self-efficacy enables individuals to believe they can effectively manage negative emotions (e.g., anxiety, anger) in social situations (M. Caprara et al., 2022). Through cognitive restructuring, interpersonal situations are reinterpreted from stressors into controllable, developmental challenges. This belief encourages individuals to view stress as an opportunity for growth (Bandura, 1997), leading them to initiate or engage in social interactions rather than avoid them. Moreover, in real interactions, high regulatory emotional self-efficacy allows individuals to employ emotion regulation strategies more flexibly and effectively, reducing the intensity and duration of negative emotions while promoting positive emotional expression (Doménech et al., 2024). Effective emotion management and cognitive restructuring thus facilitate smoother interpersonal outcomes, reduce conflicts, and represent the core psychological mechanism through which physical activity alleviates interpersonal distress.

### 4.3. The Moderating Effect of Psychological Resilience

The results showed that psychological resilience significantly predicted interpersonal distress in high school students and moderated the relationship between regulatory emotional self-efficacy and interpersonal distress (β = 0.042, *p* < 0.01). Specifically, regulatory emotional self-efficacy strongly reduced interpersonal distress at low levels of psychological resilience (β = −0.128, *p* < 0.001), but this protective effect weakened and became nonsignificant at high levels of resilience (β = −0.043, *p* = 0.137). This finding diverges from the initial promotion hypothesis of the protective factor–protective factor model, which posited that high resilience would amplify the role of emotional regulation self-efficacy. Instead, it supports the exclusion hypothesis, in which one protective factor (psychological resilience) diminishes the effect of another (regulatory emotional self-efficacy) on interpersonal distress (Bao et al., 2013).

This finding can be interpreted through triadic reciprocal determinism and the protective–protective model, particularly from the perspective of resource substitution. From the viewpoint of triadic reciprocal determinism, high psychological resilience, as a strong internal factor, reduces high school students’ reliance on regulatory emotional self-efficacy when coping with interpersonal distress. From the protective–protective model, resilience and regulatory emotional self-efficacy have overlapping functions in enhancing adaptability. As an important psychological resource, resilience not only directly buffers stress but also partially overlaps with the core function of self-efficacy by improving general adaptability, such as flexible emotional regulation (Troy et al., 2023). Resource substitution theory further suggests that when individuals possess multiple psychosocial resources with similar or substitutive functions, dependence on any single resource decreases (Ross & Mirowsky, 2006). When a core resource such as psychological resilience is lacking, other substitute resources—such as regulatory emotional self-efficacy—become crucial for coping with challenges. In this sense, a resource substitution effect emerges when protective factors with similar functions interact. Prior studies confirm that resilience enhances adolescents’ confidence in handling stress (M. Li et al., 2025). Thus, for high school students with low resilience, emotional self-efficacy functions as a key substitute resource, compensating for their lack of confidence by strengthening emotion management skills and enabling them to cope with interpersonal challenges (X. Zhang et al., 2022). By contrast, for highly resilient students, resilience itself—as a powerful and internalized core resource—helps them maintain low levels of interpersonal distress (X. Li et al., 2022). Their overall confidence in coping reduces the need for domain-specific confidence in regulatory emotional self-efficacy, rendering its additional contribution to alleviating interpersonal distress redundant and nonsignificant.

### 4.4. Theoretical Implications

This study both verified and extended social cognitive theory (triadic reciprocal determinism) and the protective–protective model by elucidating the mechanism through which physical activity alleviates interpersonal distress via enhanced regulatory emotional self-efficacy. Furthermore, it identified an innovative finding regarding the nuanced moderating role of psychological resilience within this mechanism. Specifically, psychological resilience exerted a “substitution effect” on high school students’ regulatory emotional self-efficacy—whereby high resilience, as a core personal resource, partially substitutes for the protective influence of regulatory emotional self-efficacy in reducing interpersonal distress. This finding enriches the protective–protective model by deepening our understanding of how protective factors interact, particularly in relation to the rejection hypothesis.

### 4.5. Recommendation

It is recommended that interventions for high school students with low psychological resilience focus on enhancing their regulatory emotional self-efficacy. The education department could incorporate “promoting emotional regulation skills through physical activity” into school mental health education guidelines and physical education curriculum standards. Schools may offer mindfulness-based exercise clubs (e.g., tai chi, yoga) to cultivate body awareness, breathing regulation, and confidence in managing negative emotions. Such approaches provide a cost-effective strategy to compensate for limited resilience resources and reduce interpersonal distress.

For students with high psychological resilience, merely strengthening regulatory emotional self-efficacy may have limited benefits. Schools should instead identify and nurture these students by integrating them into mental health promotion systems as peer supporters and role models, while also providing advanced and challenging activities (academic, athletic, and interpersonal) to meet developmental needs. Interventions should emphasize consolidating their psychological resilience advantages. For example, through group counseling or classroom discussions, they can be guided to share and refine the effective strategies they use to cope with academic and interpersonal challenges (such as problem solving, seeking support, and positive re-evaluation) to strengthen their adaptive coping patterns. They can also be encouraged to take on roles in sports clubs, class activities, or peer counseling to use their resilience advantages to help and support others, strengthen individual coping strategies, and promote positive cognitive patterns, thereby avoiding interpersonal distress.

### 4.6. Limitations

Although this study explored the internal mechanisms linking physical activity and interpersonal distress among high school students and yielded positive findings, several limitations remain. First, as a cross-sectional study, it can only provide predictive results based on correlational strategies, making it difficult to draw firm causal inferences. Future research should employ longitudinal designs or experimental methods to clarify causal relationships. Second, due to practical constraints, this study did not include third-year students. Given the intense pressure of the college entrance examination, their levels of interpersonal distress, physical activity, and psychological resources may differ from those of junior students. Moreover, participants were drawn only from four regions of Jiangsu Province. Considering China’s vast cultural, educational, and economic diversity, findings may not generalize nationwide. Future studies should therefore adopt broader sampling across regions and cultural contexts. Third, this study controlled only for gender, region, and family type and did not account for other potentially important confounding variables such as academic stress and socio-economic status. Future work should incorporate these measures. Finally, the IPAQ-S used to assess physical activity (MET-min/week) does not distinguish between activity types (e.g., individual vs. group). Thus, our conclusion that “physical activity, especially group activities, may reduce interpersonal distress” should be interpreted cautiously within the context of Chinese high school students’ living conditions and activity patterns.

## 5. Conclusions

Physical activity can significantly predict interpersonal distress in high school students; regulatory emotional self-efficacy mediates the relationship between physical activity and interpersonal distress; psychological resilience can play a moderating role in the relationship between regulatory emotional self-efficacy and interpersonal distress. Specifically, when high school students have low psychological resilience, their interpersonal distress will significantly decrease with the improvement of regulatory emotional self-efficacy; however, when high school students have high psychological resilience, the moderating effect is not significant.

## Figures and Tables

**Table 1:** Sample characteristics.

Characteristic	Category	n	%
Gender	Male	1071	51.6%
	Female	1004	48.4%
Grade	First-year	1011	48.7%
	Second-year	1064	51.3%
Region	North Jiangsu	537	25.9*%*
	Central Jiangsu	567	27.3*%*
	South Jiangsu	486	23.4*%*
	Nanjing	485	23.4*%*
Family type	Normal family	1929	93.0*%*
	Reconstituted family	59	2.8*%*
	Single-parent family	87	4.2*%*

**Table 2:** Psychometric Properties of Measurement Instruments.

Scale	Cronbach’s Alpha	CFA Fit Indices			
		*χ*^2^/df	CFI	TLI	RMSEA
Adolescent Psychological Resilience Scale	0.883	5.003	0.903	0.908	0.044
Regulatory Emotional Self-Efficacy Scale	0.907	5.963	0.951	0.919	0.068
Interpersonal Relationship Comprehensive Diagnostic Scale	0.852	4.061	0.887	0.869	0.039

Note: CFA = confirmatory factor analysis; *χ*^2^/df = chi-square/degrees of freedom; CFI = comparative fit index; RMSEA = root mean square error of approximation.

**Table 3:** Participants’ demographics and characteristics.

Variables	t/f-Value *p*-Value	PA	RESE	PR	IRCD
**Gender**					
Male		1822.37 ± 1566.34	45.84 ± 9.09	93.68 ± 15.45	8.40 ± 5.60
Female		1242.51 ± 1106.38	44.06 ± 8.63	94.42 ± 15.25	8.61 ± 4.94
	t-value	9.79 ***	4.57 ***	−1.09	−0.88
**Grade**					
First-year		1524.43 ± 1218.49	44.82 ± 9.14	93.96 ± 15.60	8.46 ± 5.40
Second-year		1558.31 ± 1541.82	45.12 ± 8.69	94.12 ± 15.12	8.54 ± 5.19
	t-value	−0.553	−0.76	−0.236	−0.338
**Region**					
North Jiangsu		1341.88 ± 1572.16	45.80 ± 8.57	95.80 ± 14.95	7.83 ± 5.05
Central Jiangsu		1792.78 ± 1264.98	46.78 ± 9.32	94.94 ± 15.39	8.29 ± 5.60
South Jiangsu		1547.05 ± 1238.71	43.63 ± 8.69	93.90 ± 15.76	8.90 ± 5.11
Nanjing		1464.47 ± 1431.89	43.31 ± 8.52	91.18 ± 14.97	9.08 ± 5.26
	F-value	10.46 ***	419.03 ***	6.13 ***	6.13 ***
	*p*-value	<0.001	<0.001	<0.001	<0.001
**Family type**					
Normal family		1538.18 ± 1380.39	45.12 ± 8.89	94.29 ± 15.29	8.38 ± 5.27
Reconstituted family		1608.73 ± 1580.01	43.22 ± 9.66	90.93 ± 16.37	9.81 ± 5.49
Single-parent family		1576.70 ± 1555.86	43.09 ± 8.78	90.64 ± 15.54	10.22 ± 5.29
	F-value	0.10	3.34 *	3.60 *	6.93 ***

Note: Quantitative data were expressed by “mean” and “standard deviation (SD)”; qualitative data were expressed by “n” and “percentage (%)”. PA = physical activity; RESE = regulation emotional self-efficacy; PR = psychological resilience; IRCD = interpersonal relationship distress; same as below. * means that *p*-value is <0.05; *** means that *p*-value is <0.001, same as below.

**Table 4:** Correlation between variables.

	PA	RESE	PR	IRD
1. PA	1			
2. RESE	0.15 **	1		
3. PR	0.12 **	0.56 **	1	
4. IRCD	−0.11 **	−0.37 **	−0.53 **	1

Note: All values are statistically significant at *p* < 0.01 (**).

**Table 5:** The results of the regression estimate of the mediation model (standardization).

Outcome Variable	IRCD		RESE		IRCD	
	*β*	*t*	*β*	*t*	*β*	*t*
Gender	−0.002	−0.044	−0.160	−3.645 **	−0.059	−1.404
Region (Central Jiangsu)	0.414	2.343 *	0.059	1.001	0.162	2.875 **
Region (South Jiangsu)	0.231	3.731 **	−0.272	−4.454 ***	0.135	2.306 *
Region (Nanjing)	0.242	3.911 **	−0.295	−4.828 ***	0.138	2.358 *
Family type (Blended family)	0.294	2.246 *	−0.207	−1.605	0.220	1.796
Family type (single-parent family)	0.345	3.174 **	−0.203	−1.894	0.273	2.677 **
PA	−0.118	−5.274 ***	0.122	5.534 ***	−0.075	−3.535 **
RESE					−0.355	−16.986 ***
R	0.17		0.23		0.35	
R^2^	0.03		0.05		0.15	
F	8.79		16.97		44.83	

Note: Virtual coding is used for regions and family type. * means that *p*-value is <0.05; ** means that *p*-value is <0.01; *** means that *p*-value is <0.001.

**Table 6:** Mediation Analysis: Standardized Effects and 95% Confidence Intervals.

	Effect Value	Bootstrap 95% CI		Proportion of Relative Associations
		Boot LLCI	Boot ULCI	
Total associations	−0.118	−0.162	−0.074	100%
Direct associations	−0.075	−0.116	−0.033	39.04%
Indirect associations	−0.043	−0.061	−0.027	63.19%

**Table 7:** Testing of the moderated mediation model (standardization).

Outcome Variable	RESE		IRCD	
	β	t	β	t
Gender	−0.160	−3.640 **	0.029	0.759
Region (Central Jiangsu)	0.060	1.005	0.093	1.816
Region (South Jiangsu)	−0.272	−4.446 ***	0.132	2.488 *
Region (Nanjing)	−0.295	−4.820 ***	0.070	1.310
Family type (blended family)	−0.207	−1.604	0.156	1.396
Family type (single-parent family)	−0.203	−1.893	0.223	2.415 *
PA	0.122	5.533 ***	−0.045	−2.360 *
RESE			−0.083	−3.626 **
PR			−0.478	−21.180 ***
RESE × PR			0.043	2.637 **
R	0.23		0.55	
R^2^	0.05		0.30	
F	16.95		88.20	

* means that *p*-value is <0.05; ** means that *p*-value is <0.01; *** means that *p*-value is <0.001.

**Table 8:** Conditional effects of the focal predictor at values of the moderator.

PR	Effect	t	LLCI	ULCI
−1.000	−0.127	−4.756 ***	−0.180	−0.075
0.000	−0.085	−3.753 ***	−0.130	−0.040
1.000	−0.043	−1.488	−0.100	0.014

*** means that *p*-value is <0.001.

## Data Availability

The data presented are available on request from the corresponding author.
